# Syncope: Review of Monitoring Modalities

**DOI:** 10.2174/157340308783565447

**Published:** 2008-02

**Authors:** Rajesh Subbiah, Lorne J Gula, George J Klein, Allan C Skanes, Raymond Yee, Andrew D Krahn

**Affiliations:** Division of Cardiology, University of Western Ontario, London, Ontario, Canada

## Abstract

Elucidating the underlying cause of unexplained syncope, palpitations or other possible arrhythmia-related symptoms is a formidable clinical challenge. Cardiac monitoring supplements the most important “test” in patients with syncope or palpitations, that of a thoughtful history and physical examination. Ideally, comprehensive physiologic monitoring during spontaneous symptoms would constitute what, at present, is an unattainable gold standard test for establishing a cause. Short of that goal, establishing an accurate symptom-rhythm correlation can often provide a diagnosis. Ambulatory outpatient monitoring is a powerful diagnostic tool for the evaluation of cardiac arrhythmias. Evolving technologies have provided a vast array of monitoring options for patients suspected of having cardiac arrhythmias, with each modality differing in duration of monitoring, quality of recording, convenience and invasiveness. Holter monitors, event monitors and external loop recorders are non-invasive and provide easily accessible short-term monitoring solutions. In instances where the diagnosis remains elusive, a more long-term strategy with an implantable loop recorder may be the preferred path.

## INTRODUCTION

The term syncope has its origins in ancient Greek. From an etymological viewpoint, it is composed of the prefix “syn”, meaning with or together, and the verb “copto”, meaning to cut short or interrupt. The *sine qua non* of syncope is transient loss of both consciousness and voluntary muscle tone secondary to a temporary global reduction in cerebral blood flow. Syncope is a frequently encountered clinical conundrum with an estimated lifetime prevalence of up to 35% [1]. Syncope accounts for up to 3% of emergency department consultations and 6% of hospital admissions [2-4]. Elucidating the underlying cause of unexplained syncope can pose a clinical challenge, which is difficult yet worthwhile, as identification of underlying cardiac disease in patients with syncope is associated with higher rates of mortality and morbidity [5]. Although the most important “test” in patients with syncope or palpitations remains a thorough history and physical examination [6,7], cardiac monitoring is an essential diagnostic adjunct. Choice of investigative modalities is determined by the initial clinical evaluation. For instance, patients presenting with exercise-induced syncope benefit from cardiac imaging to exclude structural disease such as a hypertrophic cardiomyopathy or coronary artery disease [8]. Further investigation with exercise stress testing can lead to the diagnosis of ischemia or arrhythmias precipitated by exercise, in contrast to post-exertional syncope that may be associated with autonomic dysfunction [2]. Comprehensive physiologic monitoring during spontaneous symptoms constitutes an often unattainable gold standard for establishing a cause. Short of that goal, establishing an accurate symptomrhythm correlation can often provide a diagnosis, making ambulatory outpatient monitoring a powerful diagnostic tool for the evaluation of cardiac arrhythmias. Evolving technologies have provided a wide array of monitoring options for patients suspected of having cardiac arrhythmias, with each modality differing in duration of monitoring, quality of recording, convenience, and invasiveness. Holter monitors, event monitors and external loop recorders are non-invasive and provide easily accessible short-term monitoring. In instances where the diagnosis remains elusive, a longer-term strategy with an implantable loop recorder (ILR) may be the preferred path.

## HOLTER MONITORING

A standard ECG should be ordered for all patients with syncope [2,9]. Short term electrocardiographic monitoring *via* three or, in some cases, twelve surface electrodes is the most common initial investigation in patients who present with syncope or palpitations. Typically this occurs in the emergency room or primary care setting with telemetry and continuous monitoring. More recently, however, wireless telemetry offers the possibility of reviewing continuous electrocardiogram recordings instantaneously at particular access points [10]. Nonetheless, the overall diagnostic yield of Holter monitoring is low. In a pooled analysis by Linzer *et al.,* among patients with symptoms of syncope or presyncope there was a 4% correlation between symptoms and arrhythmias with Holter monitoring for more than 12 hours [9].

The findings on electrocardiographic monitoring must be correlated with symptoms, as heart rate, and even cardiac rhythm, is often uninformative in the absence of clinical correlation. The major limiting factor in the diagnosis of the index event is the likelihood of another syncopal episode during the monitoring period. Holter monitoring can also be used as a strategy to exclude a significant arrhythmic cause of symptoms in the primary care setting. Presyncope is a more common event during ambulatory monitoring but is less likely to be associated with an arrhythmia [11,12]. Additionally, the ubiquitous nature of presyncope makes it a relatively poor surrogate for the assessment of syncope.

**Fig. (1). Holter Monitor.**
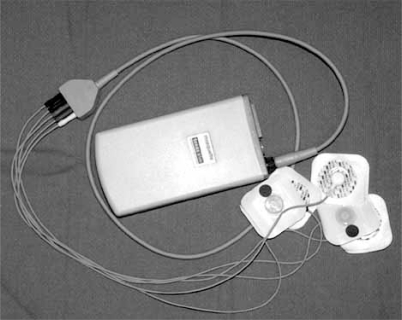
The recording device is worn by the patient using a shoulder strap or belt loop, attaching to 3-5 skin electrodes for continuous monitoring. An event button (not shown) at the top of the housing of the device is pressed in the event of symptoms to mark the recording. See text for discussion.

Holter monitoring is useful in unexplained syncope or where an arrhythmic etiology is suggested by history in a patient at relatively high risk for arrhythmia (i.e. underlying structural heart disease or abnormal baseline electrocardiogram). The Holter monitor is a portable battery-operated device that connects to the patient using electrodes, providing recordings from up to twelve electrocardiographic leads. Data are stored in the device using analog or digital storage media. The data are transformed into a digital format and analyzed using interpretive software. Additional markers for patient-activated events and time correlates are included, along with a patient event diary, to allow greater diagnostic accuracy. Continuous electrocardiographic monitoring is possible for a maximum of 48 hours (See Fig. **1**). This may allow the documentation of cardiac rhythm during symptomatic and/or asymptomatic events.

There are, however, a number of limitations with Holter monitoring. The major limitation is that patients may not experience symptoms or cardiac arrhythmias during the recording period. The physical size of the device may impair the ability of patients to sleep comfortably or engage in activities that precipitate or reproduce symptoms. Patients are further inconvenienced because the device has to be removed while showering or bathing. There is also considerable variability in patient documentation and recollection of activated events, such that accurate symptom-rhythm correlation is undermined. It is therefore not surprising that Holter monitoring has a low diagnostic yield. In several large series of patients undergoing twelve or more hours of ambulatory monitoring for investigation of syncope, only 4% had recurrence of symptoms during monitoring [9,13,14]. The overall diagnostic yield of ambulatory or Holter monitoring was 19%. Uncommon asymptomatic arrhythmias such as prolonged sinus pauses, atrio-ventricular block (such as Mobitz type II block), and non-sustained ventricular tachycardia can provide important contributions to the diagnosis, often necessitating further investigations to rule out structural heart disease and other precipitating factors. While these observations require prompt attention, it is important to interpret the results in the clinical context of the syncopal presentation so that common causes of syncope, such as neurocardiogenic syncope, are not unduly excluded.

It is also important to recognize that normal ambulatory electrocardiographic monitoring does not exclude an arrhythmic cause for syncope. If the pre-test probability is high for an arrhythmic cause, then further investigations such as prolonged monitoring or electrophysiological studies are required. One study has investigated an extended duration of ambulatory Holter monitoring of 72 hours [13]. In this study, an increased number of asymptomatic arrhythmias were detected, even though the overall diagnostic yield was not improved. The more frequent the symptoms, the higher the diagnostic yield of Holter monitoring. The apparent modest yield of Holter monitoring presumably reflects the primary care use of the device in patients with frequent symptoms, facilitating a symptom-rhythm correlation. This leads to selection bias in the referral population, as referred patients tend to have failed short term monitoring, suggesting infrequent symptoms and the need for longer-term monitoring strategies.

## EXTERNAL LOOP RECORDERS AND TRANSTELEPHONIC MONITORS

**Fig. (2). Transtelephonic Monitors.**
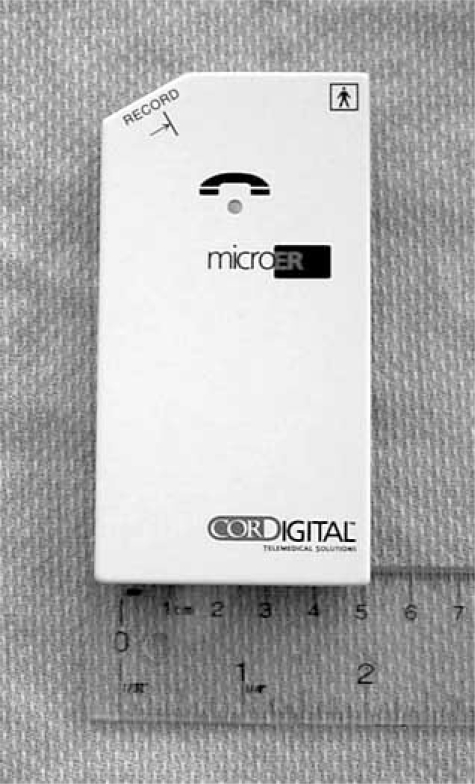
The device is lightweight and portable. Four recording electrodes are present on the back of the device to permit single lead rhythm strip capture. A record button (top left) is pressed at the onset of symptoms, and the recorded event is transmitted to a base station over an analog phone line.

Transtelephonic electrocardiographic monitors are recording devices that transmit data *via* an analog phone line to a base station (Fig. **2**). The signal is then converted to an interpretable recording that is displayed or printed as a single lead rhythm strip. There are two specific types of devices. The first does not save a recording of the rhythm for later playback and requires the patient to transmit the data to the base station, where it can be analyzed. The second type of device, with solid-state memory capacity allowing recording and storage of electrocardiographic signals during symptoms, has replaced the non-recording units. The electrocardiographic signals are collected on a real-time minute loop.

**Fig. (3). Loop Recorders.**
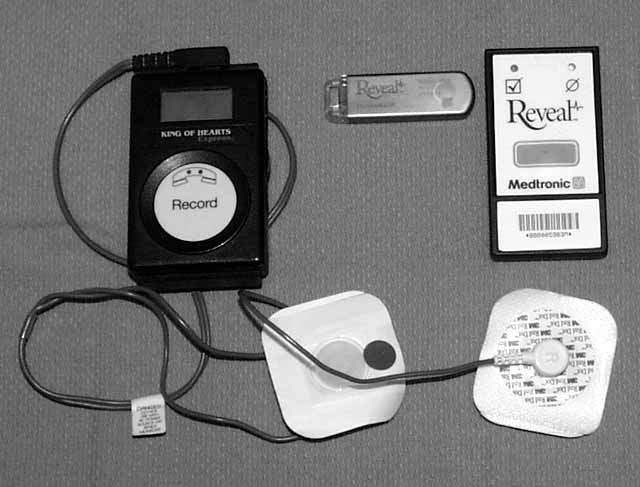
An external loop recorder (left) with cables that attach to the patient. The record button is pressed in the event of symptoms to store the previous 9 minutes, and the ensuing minute. The phone receiver is also placed over this button to transmit data over an analog phone line. An implantable loop recorder (center) and patient activator (right). The patient activator is used to “freeze” symptomatic events that are retrieved with a pacemaker programmer. Automatic events can also be captured (see text for discussion).

**Fig. (4). External Loop Recorder Tracing.**
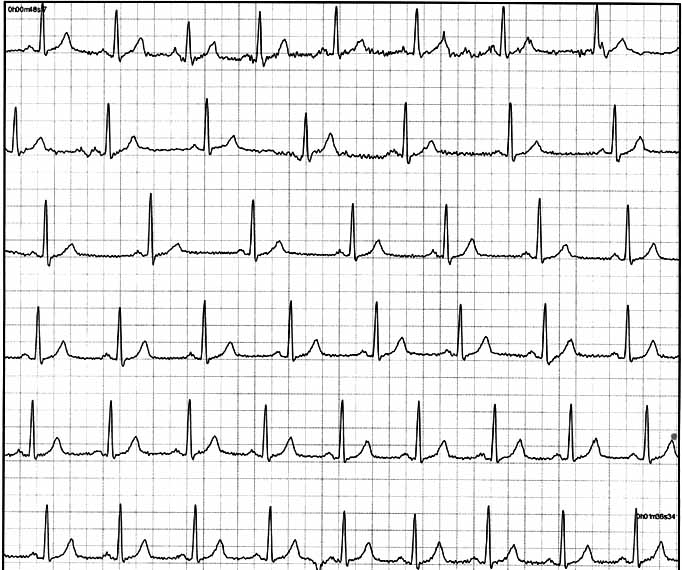
Sinus rhythm during presyncope is recorded in a 43-year-old female with recurrent unexplained syncope and presyncope. The fluctuation in heart rate is suggestive of neurocardiogenic syncope.

An external cardiac loop recorder continuously records and stores an external single modified limb lead electrogram with a 4-18 minute memory buffer (Fig. **3**, left). After the onset of spontaneous symptoms the patient activates the device, which stores the previous 3-14 minutes, and the following 1-4 minutes, of recorded information. The captured rhythm strip can subsequently be uploaded and analyzed (Fig. **4**) and often provides critical information regarding the onset of the arrhythmia. This system can be used for weeks to months provided weekly battery changes are performed. The recording device is attached with two leads to the patient’s chest wall and needs to be removed for bathing or showering, and can be uncomfortable during sleep.

Long-term compliance with this device can be challenging because of electrode and skin-related problems and waning of patient motivation in the absence of recurrent symptoms. Linzer *et al.* reported the use of patient-activated loop recorders in 57 patients with syncope and non-diagnostic findings on history, physical examination and 24 hour Holter monitoring [15,16]. A diagnosis was obtained in 14 of 32 patients who had recurrence of symptoms. Device malfunction, patient non-compliance or inability to activate the recorder was responsible for the lack of diagnosis in the remaining 18. Other studies have also reported similar findings [16,17] and demonstrated that loop recorders are complementary to 24 hour ambulatory electrocardiographic monitoring. The diagnostic yield for external loop recorders in these three studies [15-17] ranged from 24%-47%, with highest yield in patients with palpitations.

A prospective randomized clinical trial compared the utility of external loop recorders to conventional Holter monitoring in a community based referral population with syncope and presyncope [18]. Not surprisingly, the ability to obtain a symptom-rhythm correlation was 22% for Holter monitoring and 56% for the external loop recorder (p < 0.001), with a duration of monitoring of 48 hours and 4 weeks, respectively. A higher diagnostic yield was also obtained among patients randomized to Holter monitoring who remained undiagnosed and crossed-over to use of a loop recorder. This trial suggests that loop recorders should be considered as first line monitoring when attempting to establish a symptom rhythm correlation in the initial workup of patients with syncope. Twenty-four percent of loop recorder patients failed to activate the device properly, suggesting limited usefulness in some patients [18]. Analysis of factors pertaining to use of external loop recorders has revealed a particularly low diagnostic yield among patients who are unfamiliar with technology, live alone, or have low motivation for achieving a diagnosis [19]. Reiffel *et al*. [20] retrospectively compared the results obtained by Holter monitoring, loop recording and auto-triggered loop recording in 600 patients from a database of approximately 100,000 patients. The auto-triggered loop recording approach provided a higher yield of diagnostic events (36%) compared to loop recording (17%) and Holter monitoring (6.2%).

The external loop recorder appears to have its greatest role in motivated patients with frequent spontaneous symptoms that are likely to recur within 4-6 weeks. Given that it is non-invasive and cost effective, loop recording should be considered in all patients in whom an arrhythmic cause for syncope is suspected, keeping in mind that the major limitation of this device is the need to continuously wear external electrodes.

## IMPLANTABLE LOOP RECORDERS

The implantable loop recorder (ILR) is a relatively recent investigational tool in undiagnosed syncope that permits prolonged monitoring without external electrodes. It is ideally suited to patients with infrequent recurrent syncope thought to be due to an arrhythmic cause. Similar to the external loop recorder, it is designed to correlate physiology with recorded cardiac rhythms, but implanted and therefore devoid of surface electrodes and accompanying compliance issues. The ILR also monitors much longer time periods than an external loop recorder. Currently the only FDA approved ILR (Medtronic Reveal Plus^®^ Model 9526) has a pair of sensing electrodes with 3.7 cm spacing on a small elongated recording device 6 cm long, 2 cm wide 0.8 cm thick, and weighing 17 grams (Fig. **3**, center). The battery life is 18-24 months. The device can be implanted subcutaneously in the chest wall under local anesthetic and with antibiotic prophylaxis.

Prior to implantation, cutaneous mapping should be performed to optimize the sensed signal and avoid T-wave oversensing, which can be falsely interpreted as a high rate episode. An adequate signal can usually be obtained anywhere in the left hemithorax [21]. The recorded bipolar signal is stored in the device as 21 minutes of uncompressed or 42 minutes of compressed signal. A compressed signal is generally used with only marginal loss of quality while maximizing memory capability. The patient, along with a spouse, family member or friend is instructed in the use of the activator at the time of implant. Once an episode is recorded (i.e. a presyncopal or syncopal event occurs) the memory is “frozen” by the patient or a relative using a non-magnetic hand held activator (Fig. **3**, right). The episode is then uploaded for interrogation to a pacemaker programmer (Medtronic 9790, Medtronic, Minneapolis, Minnesota, U.S.). Although heart rate is usually easily ascertained, p waves can occasionally be challenging to interpret. The current version of the ILR has programmable automatic detection of rapid and slow heart rate episodes as well as pauses.

**Fig. (5). Automatic Event Detection from an ILR.**
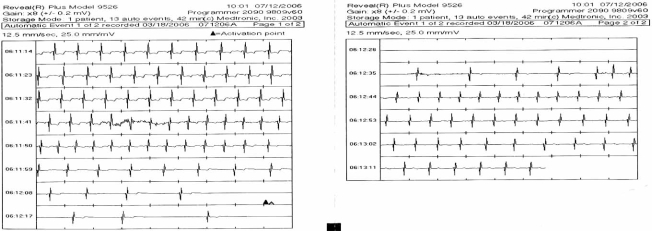
This is a typical tracing of an event captured by an ILR during syncope in a patient. The arrow and letter A denotes automatic activation when the device detects a 3 second pause. Each line constitutes 10 seconds of a single lead rhythm strip. Note the slowing of the sinus rate prior to onset of a prolonged pause, which resulted in syncope. This is consistent with the diagnosis of neurocardiogenic syncope (ISSUE classification 1A).

**Fig. (6). Manual Event Detection from an ILR.**
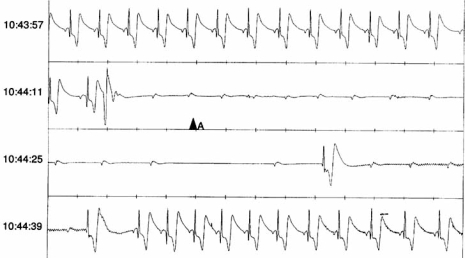
Manual activation during presyncope in a 73-year-old male with two previous episodes of unexplained syncope. There is subsequent sinus showing suggests a secondary vasovagal response.This is classified as a 1C response by the proposed ISSUE classification, suggesting intrinsic AV node disease.

A classification system for recorded events has been proposed by Brignole *et al.* [22] (Table **1**) that categorizes the probable mechanism of syncope according to the pattern of bradycardia recorded during spontaneous episodes. An example of the cardioinhibitory component of neurocardiogenic or vasovagal syncope is illustrated in Fig. **5**. This would be considered a 1A response. Fig. **6** illustrates a primary bradycardia (1C response), highly suggestive of intrinsic AV node disease. This classification is useful for research purposes for event classification, and is likely to prove useful in directing therapy once validated.

Currently there are several studies establishing the utility of ILR in the diagnosis of syncope [22-27]. One of these studies is a multi-centre study of 206 patients [26]. The majority of patients had undergone non-invasive and invasive testing including head-up tilt testing and electrophysiological studies. The etiology of syncope was arrhythmic in 22% of patients. Fourty-two percent had further symptoms with only sinus rhythm documented, and 22% had no further episodes [26]. Bradycardia was the most commonly detected arrhythmia (17% *vs*. 6% tachycardia), usually leading to pacemaker implantation [26]. From this study, 4% of patients failed to properly activate the device and thus did not establish a symptom rhythm correlation. The devices used in this study did not contain the automatic detection feature. Multivariate modeling did not identify any significant pre-implant predictors of subsequent arrhythmia detection other than a weak association with advancing age and bradycardia. No age group had an incidence of bradycardia greater than 30% [26].

In a group of patients with ongoing seizures despite anticonvulsant therapy, Zaidi *et al.* performed cardiac assessment including head-up tilt testing and carotid sinus massage in all patients, and implantation of an ILR in ten patients [28,29]. Two of the ten patients with an ILR had marked bradycardia preceding a seizure; one due to sinus pauses and the other due to heart block. Importantly, this study suggested seizures that are atypical in presentation may have a cardiovascular cause in as many as 42% of cases, and cardiovascular assessment including long term cardiac monitoring with an ILR may play a role in select patients with atypical seizures.

In three studies [2,30,31] from the International Study on Syncope of Uncertain Etiology (ISSUE) investigators, ILRs were implanted in different groups of patients with syncope to assess cardiac rhythm during episodes, after conventional testing. The first study involved tilt tests in 111 patients with unexplained syncope, and loop recorders implanted after the tilt test, regardless of result [30]. Syncope recurred in 34% of patients in both the tilt positive and tilt negative group, with marked bradycardia or asystole the most commonly recorded arrhythmia during follow-up (46% and 62% respectively). The heart rate during tilt testing did not predict spontaneous heart rate response, with a much higher incidence of asystole than expected based on demographics or tilt. This study suggests that observations during tilt testing correlate poorly with cardiac rhythm during spontaneous syncope, and that bradycardia is more common in this population than previously recognized. This was observed in ISSUE 2, where approximately 12% (47/392 patients) had asystole documented as the cause of recurrent syncope [32]. An ILR study of symptomatic vasovagal patients also showed that the heart rhythm observed during a spontaneous syncope did not correlate with the head-up tilt test [33]. An example of the cardioinhibitory component of vasodepressor syncope is illustrated in Fig. **5**.

In the second study, 52 patients with syncope, bundle branch block and negative electrophysiologic testing underwent ILR implantation [2]. Syncope recurred in 22 of the 52 patients with conduction system disease. Long term monitoring demonstrated marked bradycardia mainly attributed to complete AV block in 17, while it excluded AV block in 2. Three patients did not properly activate the device after symptoms. This study confirmed that negative electrophysiologic testing does not exclude intermittent complete AV block, and that prolonged monitoring or consideration of permanent pacing is reasonable in this population.

The third study examined the spontaneous rhythm in 35 patients with syncope, overt heart disease and negative electrophysiologic testing [31]. The underlying heart disease was predominantly ischemic or hypertrophic cardiomyopathy with moderate left ventricular dysfunction. Although previous studies have suggested that patients with negative electrophysiologic testing have a better prognosis, there remains concern regarding risk of ventricular tachycardia in this group. Symptoms recurred in 19 of the 35 patients (54%), with bradycardia in 4, supraventricular tachyarrhythmias in 5 and ventricular tachycardia in only 1 patient. There were no sudden deaths during 16 ±11 months of follow-up.

A prospective randomized trial compared early use of the ILR for prolonged monitoring to conventional testing in patients undergoing a cardiac workup for unexplained syncope (Randomized Assessment of Syncope Trial – RAST) [24, 34]. Sixty patients (age 66 ±14 years) with unexplained syncope were randomized to “conventional” testing with an external loop recorder, tilt test and electrophysiologic study versus one year of monitoring with an implantable loop recorder. Patients were excluded if they had a left ventricular ejection fraction less than 35%. If patients remained undiagnosed after their assigned strategy, they were offered crossover. A diagnosis was obtained in 14 of 27 patients randomized to prolonged monitoring, compared to 6 of 30 undergoing conventional testing (52% *vs*. 20%, p=0.012). Overall, prolonged monitoring was more likely to result in a diagnosis than conventional testing (55% *vs*. 19%, p=0.0014). Bradycardia was detected in 14 patients undergoing monitoring, compared to 3 patients with conventional testing (40% *vs*. 8%, p=0.005). These data highlight the diverse etiology of syncope, and also illustrate the limitations of conventional diagnostic techniques. Although there is clear selection bias in enrollment of patients referred to an electrophysiologist for workup, this study suggests that tilt testing has a modest yield when applied to all patients undergoing investigation for unexplained syncope, and that electrophysiologic testing is of very limited utility in patients with preserved left ventricular function. Also in patients with a negative electrophysiology study for suspected arrhythmia tilt testing has been shown to be of little value in predicting the mechanism of syncope [35].

A recent prospective, multicenter observational study (ISSUE 2) investigated the efficacy of therapies based on ILR diagnosis of recurrent suspected neurocardiogenic syncope [32]. Patients were included in the study if they experienced three or more clinically severe syncopal episodes over 2 years without significant electrocardiographic or cardiac abnormalities. Those with postural hypotension and carotid sinus syncope were excluded. After the first documented episode of syncope after ILR implantation, the device was interrogated and therapy was prescribed accordingly. The 1-year recurrence rate of syncope in 392 patients was 33%. Among 103 patients with a documented episode, 53 patients received loop recorder directed therapy; 47 receiving a pacemaker due to asystole and 6 receiving anti-tachyarrhythmia therapy (catheter ablation: four, implantable defibrillator: one, anti-arrhythmic drug: one). The remaining 50 patients did not receive specific therapy. The 1-year recurrence rate among the 53 patients assigned to a specific therapy was 10% compared with 41% in the patients without specific therapy. The 1-year recurrence rate in patients with pacemakers was 5%. The authors concluded that a strategy based on diagnostic information from early ILR implant, with therapy delayed until documentation of syncope, allows safe, specific, and effective therapy in patients with neurocardiogenic syncope.

## STRATEGIES FOR CHOOSING PROLONGED MONITORING

The literature clearly supports the use of the implantable loop recorder in patients with recurrent unexplained syncope that have failed a non-invasive workup and continue to have episodes. This represents a select group that has been referred for further testing, where ongoing symptoms are likely and a symptom-rhythm correlation is a feasible goal. Widespread early use of the ILR is likely to result in a poor diagnostic yield, as an increased proportion of patients will not have arrhythmia, supported by data from the RAST trial [24,34]. The optimal patient for prolonged monitoring with an external or implantable loop recorder has symptoms suspicious for arrhythmia; namely abrupt onset with minimal prodrome, typically brief loss of consciousness and complete resolution of symptoms within seconds to minutes. ISSUE 2 suggests that documentation of the cardioinhibitory component of vasovagal syncope may identify a group of patients that respond well to pacing. Brignole and colleagues addressed this hypothesis, by evaluating the effect of placebo pacing therapy [36]. Syncope recurred in 38% of patients randomized to placebo *vs*. 34% randomized to no treatment. The recurrence rate with active cardiac pacing was 15%. The authors suggest that the use of specific selection criteria for pacing, such as characteristics of the observed cardioinhibitory reflex may identify those who will respond to cardiac pacing [36].

After clinical assessment, including assessment of left ventricular function, a decision must be made if the underlying condition is potentially life threatening. All reports using the ILR have suggested a low incidence of life-threatening arrhythmia or significant morbidity with a prolonged monitoring strategy. This suggests a good prognosis for patients with recurrent unexplained syncope in the absence of left ventricular dysfunction or with negative electrophysiologic testing. This finding was particularly striking in the negative electrophysiologic testing arm of the ISSUE study (see discussion above).

Lastly, syncope fails to recur during long term monitoring in almost one third of patients even in the presence of frequent episodes prior to loop recorder implantation. This suggests that the cause of syncope in some instances is self-limited, reflecting a transient physiologic abnormality. Long term monitoring strategies may also have a role in the assessment of patients who have infrequent palpitations but are at risk of arrhythmias.

## CONCLUSION

Syncope, although relatively common, remains a significant diagnostic challenge for clinicians, despite advances in knowledge pertaining to mechanism. A careful history and examination are mandatory in the assessment of these patients, as syncope needs to be differentiated from other causes of loss of consciousness. The ultimate diagnostic goal is to correlate symptoms to rhythm disturbances, and accurate attainment of this goal requires the judicious use of monitoring strategies. Ambulatory cardiac monitoring has provided a powerful means to elucidate etiology of presyncope or syncope. The choice of ambulatory monitoring strategies is governed by index of suspicion of cardiac arrhythmias, frequency and nature of symptoms and accuracy of the monitoring device. For instance, implantable loop recorders have significantly improved the success of obtaining electrocardiographic rhythm data during spontaneous symptoms in patients with recurrent unexplained syncope. The clinician should consider early use of external and implantable loop recorders when an arrhythmia is suspected based on clinical presentation and initial non-invasive testing.

## Figures and Tables

**Table 1.** ISSUE Classification of Detected Rhythm from the ILR

Classification	Sinus Rate	AV Node	Comment	Presumed Mechansim
**Asystole (RR>3 sec)**				
1A	Arrest	Normal	Progressive sinus bradycardia with sinus arrest:	vasovagal
1B	Bradycardia	AV block	AV block with associated sinus bradycardia:	vasovagal
1C	Normal or tachycardia	AV block	Abrupt AV block without sinus slowing	intrinsic AV node disease
**Bradycardia**				
2A	Decrease>30%	Normal		vasovagal
2B	HR<40 for >10 seconds	Normal		vasovagal
**Minimal HR change**				
3A	<10% variation	Normal	Suggests unlikely vasovagal	non-cardiac cause
3B	HR increase or decrease 10-30%, not <40 or >120 bpm	Normal		vasovagal
**Tachycardia**				
4A	Progressive tachycardia	Normal	Sinus acceleration typical	orthostatic intolerance or non-cardiac cause
4B	N/A	Normal	Atrial fibrillation	Mixed – may be a component of vasovagal as well
4C	N/A	Normal	Supraventricular tachycardia	
4D	N/A	Normal	Ventricular tachycardia	

HR – heart rate, N/A – not applicable

Adapted from Brignole M, Moya A, Menozzi C, Garcia-Civera R, Sutton R. Proposed electrocardiographic classification of spontaneous syncope documented by an implantable loop recorder. *Europace.* Jan 2005;7(1):14-18 with permission.
